# CARE BASED ON PREFERENCES: EXPERIENCES OF RESIDENTS

**DOI:** 10.1093/geroni/igz038.287

**Published:** 2019-11-08

**Authors:** Martina Roes, Daniel Purwins, Tobias Stacke, Christina Manietta, Johannes Bergmann

**Affiliations:** 1 German Center for Neurodegenerative Diseases (DZNE), DZNE, Germany; 2 DZNE, Witten, Nordrhein-Westfalen, Germany

## Abstract

To provide person-centered care, professional caregivers need to know about the individual preferences of the persons being cared for. Since there were no comparable instruments available, we translated the PELI (Preferences for Everyday Living Inventory) and tested the culturally translated version in German nursing homes. Besides testing for reliability and feasibility in German care settings, were are asking for satisfaction in fulfilling of the preferences and reasons for personal or institutional barriers that hinder adherence to the preferences. Furthermore, to determine the level of understanding and meaning of preferences, we interview a few residents and their close relatives in a cognitive interview. Preliminary results of the perspective of the care recipient will be presented.

